# Liv-52 Attenuates Erlotinib-Induced Liver Injury via Modulation of Oxidative Stress, Inflammation, and Apoptosis in Rats

**DOI:** 10.3390/ijms27093817

**Published:** 2026-04-25

**Authors:** Seval Bulut, Durdu Altuner, Bahadir Suleyman, Renad Mammadov, Mustafa Ozkaraca, Ali Gungor, Mehmet Kuzucu, Engin Hendem, Halis Suleyman

**Affiliations:** 1 Department of Pharmacology, Faculty of Medicine, Erzincan Binali Yıldırım University, Erzincan 24100, Turkey; 2 Department of Pathology, Faculty of Veterinary Medicine, Sivas Cumhuriyet University, Sivas 58070, Turkey; 3 Laboratory Technician and Veterinary Health Program, Vocational School of Health Services, Osmaniye Korkut Ata University, Osmaniye 80000, Turkey; 4 Department of Molecular Biology, Faculty of Arts and Sciences, Erzincan Binali Yıldırım University, Erzincan 24100, Turkey; 5 Department of Medical Oncology, Mengucek Gazi Education and Research Hospital, Erzincan Binali Yıldırım University, Erzincan 24100, Turkey

**Keywords:** erlotinib, Liv-52, melatonin, apoptosis, inflammation, antioxidant

## Abstract

Erlotinib, an epidermal growth factor receptor tyrosine kinase inhibitor (EGFR-TKI), is widely used in cancer therapy; however, hepatotoxicity limits its clinical use. This study investigated the protective effects of Liv-52, a polyherbal hepatoprotective formulation, against erlotinib-induced hepatotoxicity in rats and compared its efficacy with melatonin. The animals (*n* = 24, Wistar albino rats) were randomly categorized into four groups: healthy (HG), erlotinib (ERG), Liv-52 + erlotinib (LEG), and melatonin + erlotinib (MEG). Liv-52 (50 mg/kg/day, orally) and melatonin (10 mg/kg/day, orally) were administered once daily for two weeks. Erlotinib (10 mg/kg, orally) was given every other day to ERG, LEG, and MEG groups for two weeks. Serum alanine aminotransferase (ALT), aspartate aminotransferase (AST), and lactate dehydrogenase (LDH) were measured. Hepatic malondialdehyde (MDA), total glutathione (tGSH), superoxide dismutase (SOD), and catalase (CAT) levels were analyzed. Additionally, double immunofluorescence staining was performed to evaluate apoptotic (poly[ADP-ribose] polymerase-1 [PARP-1], apoptosis-inducing factor [AIF]), inflammatory (cyclooxygenase-2 [COX-2]), and anti-inflammatory (interleukin-10 [IL-10]) biomarkers in liver tissues. Histopathological examination was also conducted to assess structural alterations. Erlotinib significantly increased MDA, ALT, AST, and LDH while decreasing tGSH, SOD, and CAT (*p* < 0.001). Strong immunoreactivity for PARP-1, AIF, IL-10, and COX-2, as well as severe hydropic degeneration and necrosis, was observed in ERG (*p* < 0.05). Both Liv-52 and melatonin significantly ameliorated biochemical, histopathological, apoptotic, and inflammatory alterations (*p* < 0.05). Notably, Liv-52 demonstrated superior hepatoprotective efficacy compared to melatonin. These findings indicate that Liv-52 effectively attenuates erlotinib-induced hepatotoxicity by modulating oxidative stress, inflammatory responses, and apoptotic pathways, thereby preserving liver function and structural integrity.

## 1. Introduction

Erlotinib is an anticancer agent that acts as a selective inhibitor of epidermal growth factor receptor (EGFR) tyrosine kinase activity [1]. Erlotinib has been approved by the United States Food and Drug Administration (FDA) for the treatment of advanced or metastatic non-small cell lung cancer and pancreatic cancer [2]. Erlotinib mediates its antitumor effects through inhibition of cancer cell growth, interruption of cell cycle progression, and activation of apoptosis [3]. However, erlotinib therapy may also be associated with adverse effects, including skin rash, diarrhea, and renal, hepatic, and ocular toxicities [4]. Notably, cases of severe hepatotoxicity leading to treatment interruption or discontinuation have been reported during erlotinib use [5]. Preventing erlotinib-induced hepatotoxicity is therefore clinically important, as it may directly affect treatment continuity and patient survival [6,7]. Existing trials have demonstrated that increased production of reactive metabolites plays a central role in hepatotoxicity associated with EGFR tyrosine kinase inhibitors (EGFR-TKIs) [5]. In addition, erlotinib has been associated with increased generation of reactive oxygen species (ROS) and the subsequent induction of oxidative stress in healthy tissues [8]. Excessive ROS production leads to cellular injury by damaging critical biomolecules such as DNA and proteins, contributing to both the antitumor efficacy and toxic effects of erlotinib [8,9]. Moreover, excessive ROS generation is known to trigger cell death through the activation of apoptotic signaling pathways, particularly poly(ADP-ribose) polymerase-1 (PARP-1) [10]. DNA damage induced by oxidative stress results in the overactivation of PARP-1 [10,11]. This process may contribute to mitochondrial AIF release followed by its movement to the nucleus, ultimately resulting in chromatin fragmentation and cellular demise [11]. In vitro studies have shown that erlotinib induces apoptosis in normal hepatocytes, as evidenced by alterations in nuclear morphology and increased PARP expression, ultimately resulting in hepatotoxicity [12]. In addition to oxidative stress and apoptosis, inflammatory responses have also been implicated in erlotinib-induced toxicity [8,13]. Both increased ROS production and PARP-1 overactivation have been shown to amplify inflammatory responses by upregulating proinflammatory cytokine expression [8,14,15]. A recent study further highlighted the close association between oxidative stress and inflammation in erlotinib-induced optic nerve injury [8]. Collectively, available evidence suggests that oxidative stress, inflammation, and the activation of apoptotic signaling pathways may contribute to the pathogenesis of erlotinib-induced hepatic injury.

Liv-52 is an Ayurvedic formulation that has long been used in the prevention and treatment of various liver-related conditions, including viral hepatitis, early-stage cirrhosis, protein–energy malnutrition, and loss of appetite; however, its potential protective effect against erlotinib-induced hepatotoxicity is investigated in the present study [16]. Liv-52 is a polyherbal hepatoprotective formulation containing *Capparis spinosa*, *Cichorium intybus*, *Mandur bhasma*, *Solanum nigrum*, *Terminalia arjuna*, *Cassia occidentalis*, *Achillea millefolium*, and *Tamarix gallica* [16,17]. Hepatoprotective agents are known to improve liver function, promote hepatocellular regeneration, and facilitate hepatic detoxification processes [18]. Numerous studies have demonstrated the hepatoprotective, antioxidant, anti-inflammatory, and anti-apoptotic effects of Liv-52 and its bioactive constituents [19,20]. One such study reported that Liv-52 protected liver tissues against oxidative toxicity by suppressing malondialdehyde (MDA) accumulation, preventing the depletion of endogenous antioxidants such as glutathione (GSH), superoxide dismutase (SOD), and catalase (CAT), and attenuating apoptosis [20]. Clinically, Liv-52 administration has been shown to improve biochemical and clinical parameters in various liver diseases without causing significant adverse effects [18].

Melatonin, a molecule whose hepatoprotective effects have been demonstrated in various experimental models of liver injury, has also been investigated for its protective effects against erlotinib-induced hepatotoxicity [20,21]. Melatonin is a hormone primarily produced and released by the pineal gland, and its primary physiological function is the regulation of circadian rhythms [22]. In addition to this role, melatonin is a well-known endogenous antioxidant and exhibits various biological properties, including anti-inflammatory, anti-apoptotic, and immunomodulatory effects [22,23,24]. In a preclinical study, melatonin was shown to exert hepatoprotective effects against alcoholic liver injury by reducing oxidative stress, inflammatory responses, and apoptosis [21]. In another experimental study, melatonin provided protection against hepatotoxicity induced by antituberculosis treatment through its antioxidant and anti-apoptotic actions [20]. Collectively, this evidence suggests that Liv-52 and melatonin may be beneficial in erlotinib-induced hepatotoxicity. Accordingly, this study investigated the potential hepatoprotective role of Liv-52 in rats with erlotinib-induced liver injury and compared its efficacy with melatonin.

## 2. Results

### 2.1. Evaluation of Lipid Peroxidation in Hepatic Tissues

**Figure 1:**
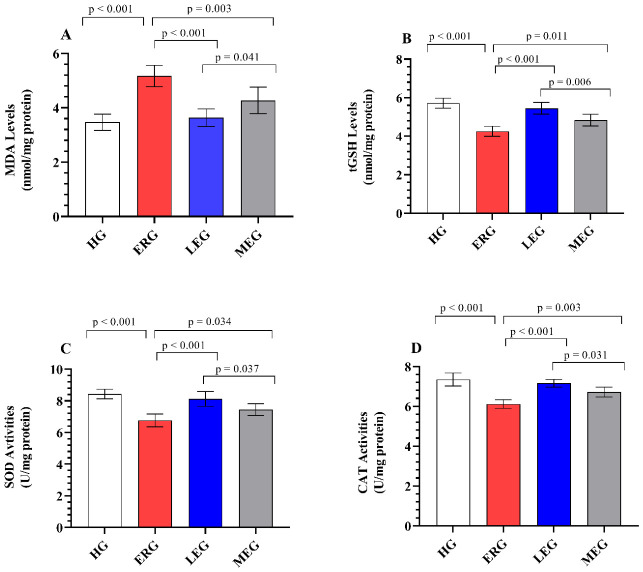
Evaluation of oxidants and antioxidants in liver tissues of experimental groups. (**A**) MDA; (**B**) tGSH; (**C**) SOD; (**D**) CAT. The bars indicate mean ± standard deviation, *n* = 6/each group. MDA and GSH levels, as well as SOD and CAT activities, were measured using rat enzyme-linked immunosorbent assay (ELISA) kits. MDA: malondialdehyde; tGSH: total glutathione; SOD: superoxide dismutase; CAT: catalase; HG: healthy group; ERG: erlotinib group; LEG: Liv-52 + erlotinib group; MEG: melatonin + erlotinib group. For statistical analysis, one-way ANOVA followed by the Tukey HSD test was used. A significance level of *p* < 0.05 was set.

Figure 1A presents the effects of the treatment groups on lipid peroxidation levels in liver tissues. Hepatic MDA levels were significantly elevated in the ERG (5.17 ± 0.40) compared with the HG (3.47 ± 0.30) (*p* < 0.001), indicating a pronounced increase in lipid peroxidation and membrane oxidative damage following erlotinib exposure. In contrast, MDA levels were significantly reduced in both the LEG (3.63 ± 0.33, *p* < 0.001) and the MEG (4.28 ± 0.49, *p* = 0.003) relative to the ERG, suggesting that both treatments attenuate lipid peroxidation and preserve membrane integrity, albeit to different extents. Notably, Liv-52 treatment attenuated the erlotinib-induced increase in MDA levels more effectively than melatonin (*p* = 0.041), highlighting its superior capacity to suppress lipid peroxidation. Furthermore, no statistically significant difference was observed between the HG and LEG (*p* = 0.874), indicating that Liv-52 nearly normalized lipid peroxidation levels to those observed under physiological conditions.

### 2.2. Evaluation of tGSH Levels in Hepatic Tissues

Figure 1B illustrates the effects of the experimental treatments on tGSH levels in liver tissues. Hepatic tGSH levels were markedly reduced in the ERG (4.26 ± 0.26) compared with the HG (5.72 ± 0.26) (*p* < 0.001), reflecting a substantial depletion of endogenous antioxidant reserves and disruption of redox homeostasis following erlotinib exposure. This erlotinib-induced depletion of tGSH was significantly alleviated by both Liv-52 (5.45 ± 0.31; *p* < 0.001) and melatonin (4.83 ± 0.30; *p* = 0.011), indicating a restoration of antioxidant capacity, albeit with differential efficacy. Notably, tGSH levels were better preserved in the LEG than in the MEG (*p* = 0.006), underscoring the superior efficacy of Liv-52 in maintaining GSH homeostasis. Furthermore, tGSH levels in the LEG were comparable to those in the HG, with no statistically significant difference observed (*p* = 0.390), suggesting a near-complete normalization of intracellular tGSH levels.

### 2.3. Evaluation of SOD and CAT Enzyme Activity in Hepatic Tissues

As shown in Figure 1C,D, hepatic SOD and CAT activities were significantly reduced in the ERG (6.77 ± 0.41 and 6.12 ± 0.22, respectively) compared with the HG (8.43 ± 0.31 and 7.35 ± 0.33, respectively) (*p* < 0.001), indicating a marked impairment in enzymatic antioxidant defense systems under erlotinib-induced stress. In contrast, the erlotinib-associated suppression of SOD and CAT activities was significantly mitigated in both the LEG (8.12 ± 0.47 and 7.16 ± 0.19, respectively; *p* < 0.001) and the MEG (7.45 ± 0.37 and 6.72 ± 0.25, respectively; *p* < 0.05) relative to the ERG, reflecting a partial to substantial reactivation of antioxidant enzyme function. This restorative effect was more prominent in the LEG than in the MEG (*p* < 0.05), suggesting a stronger capacity of Liv-52 to enhance enzymatic resilience against oxidative challenge. Furthermore, the LEG exhibited SOD (*p* = 0.519) and CAT (*p* = 0.565) activities comparable to those of the HG, indicating effective recovery of antioxidant enzyme activity to near-normal physiological conditions.

### 2.4. Serum Markers of Hepatocellular Damage

**Figure 2:**
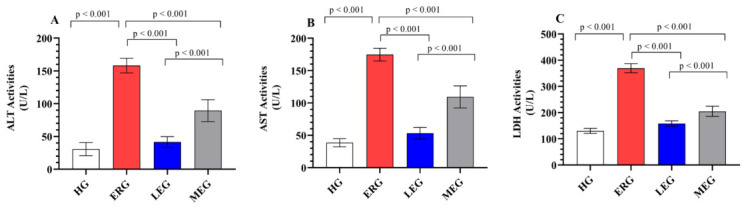
Evaluation of ALT, AST, and LDH activities in the serum of experimental groups. (**A**) ALT; (**B**) AST; (**C**) LDH. The bars indicate mean ± standard deviation, *n* = 6/each group. Serum ALT, AST, and LDH activities were determined using an automated AU5800 chemistry analyzer (Beckman Coulter) employing kinetic ultraviolet methods. ALT: alanine aminotransferase; AST: aspartate aminotransferase; LDH: lactate dehydrogenase; HG: healthy group; ERG: erlotinib group; LEG: Liv-52 + erlotinib group; MEG: melatonin + erlotinib group. For statistical analysis, one-way ANOVA followed by the Tukey HSD test was used. A significance level of *p* < 0.05 was set.

Figure 2A–C illustrate the effects of the treatment protocols on hepatocellular injury markers. ALT, AST, and LDH activities were markedly elevated in rats treated with erlotinib alone (158.17 ± 11.30, 174.67 ± 9.97, and 369.50 ± 17.30, respectively) compared with the HG (30.67 ± 9.91, 38.50 ± 6.25, and 130.17 ± 9.75, respectively) (*p* < 0.001), indicating substantial hepatocellular injury and membrane leakage induced by erlotinib. This elevation was significantly attenuated in both the LEG (41.50 ± 8.26, 53.17 ± 9.11, and 158.00 ± 10.49, respectively; *p* < 0.001) and the MEG (89.33 ± 16.74, 109.33 ± 17.12, and 204.83 ± 19.39, respectively; *p* < 0.001) relative to the ERG, reflecting a marked reduction in hepatic enzyme release and improved cellular integrity. Notably, Liv-52 administration was more effective than melatonin in suppressing these enzyme activities (*p* < 0.001), suggesting a stronger hepatoprotective capacity. Furthermore, ALT (*p* = 0.147) and AST (*p* = 0.420) levels in the LEG were comparable to those in the HG, indicating that Liv-52 largely restored hepatocellular function toward normal functional status.

### 2.5. Double Immunofluorescence Staining Results

**Figure 3:**
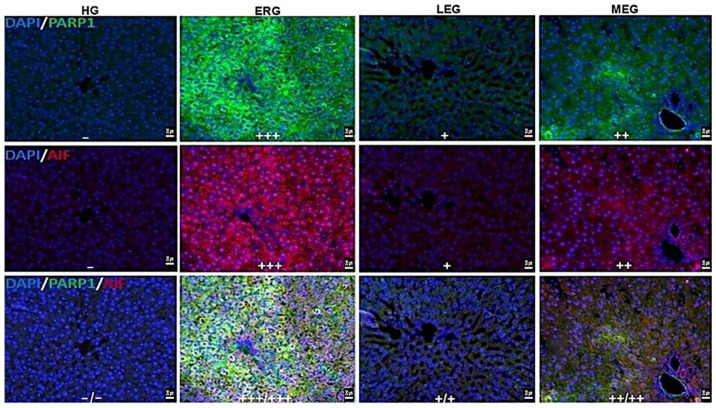
PARP-1 and AIF fluorescence positivity in liver tissues of experimental groups. Absent (−), mild (+), moderate (++), severe (+++), PARP1; FITC, AIF; Alexa Fluor 595, DAPI; 4′,6-diamidino-2-phenylindole. HG: healthy group; ERG: erlotinib group; LEG: Liv-52 + erlotinib group; MEG: melatonin + erlotinib group (Bar 20 µm).

Figure 3 and Table 1 illustrate the effects of the treatment protocols on the immunopositivity of apoptotic markers, PARP-1 and AIF, in liver tissues. liver tissues from the HG exhibited no immunofluorescence reactivity for PARP-1 or AIF, indicating the absence of basal activation of apoptosis-related pathways under physiological conditions. In contrast, the ERG demonstrated severe immunopositivity for both PARP-1 and AIF, reflecting robust activation of DNA damage-associated cell death signaling following erlotinib exposure. In the LEG, PARP-1 and AIF immunoreactivity was mild, suggesting a marked attenuation of apoptotic signaling, whereas in the MEG, immunopositivity was moderate, indicating only partial suppression of these pathways.

**Figure 4:**
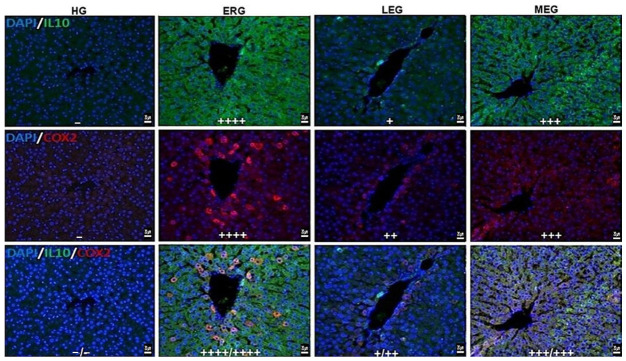
IL-10 and COX-2 fluorescence positivity in liver tissues of experimental groups. None (−), mild (+), moderate (++), severe (+++), very severe (++++). IL-10; FITC, COX-2; Alexa Fluor 595, DAPI; 4′,6-diamidino-2-phenylindole. HG: healthy group; ERG: erlotinib group; LEG: Liv-52 + erlotinib group; MEG: melatonin + erlotinib group (Bar 20 µm).

Figure 4 illustrates the effects of the treatment protocols on the immunopositivity of inflammatory (COX-2) and anti-inflammatory (IL-10) markers in liver tissues. No immunofluorescence reactivity for IL-10 or COX-2 was observed in the liver tissues of the HG, indicating a quiescent inflammatory state. Conversely, ERG showed strong immunopositivity for both anti-inflammatory (IL-10) and inflammatory (COX-2) markers. In the LEG, mild IL-10 immunoreactivity and moderate COX-2 immunoreactivity were determined, suggesting a partial modulation of the inflammatory milieu. In the MEG, severe immunopositivity was evident for both IL-10 and COX-2, indicating a limited efficacy in controlling inflammation compared with Liv-52.

### 2.6. Histopathological Findings

**Figure 5:**
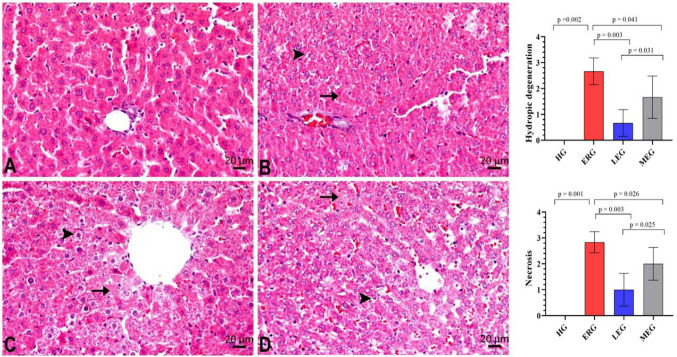
Histopathological evaluation of liver tissues from experimental groups (hematoxylin and eosin staining). (**A**). HG; Normal histological appearance. (**B**). ERG; Mild hydropic degeneration (→) with severe necrosis (►), (**C**). LEG; Mild hydropic degeneration (→) with necrosis (►), (**D**). MEG; Moderate hydropic degeneration (→) with necrosis (►). HG: healthy group; ERG: erlotinib group; LEG: Liv-52 + erlotinib group; MEG: melatonin + erlotinib group.

Figure 5 and Table 2 present the histopathological changes in liver tissues among the experimental groups. The HG showed preserved hepatic architecture without any evidence of pathological alterations. In contrast, liver tissues from rats treated with erlotinib alone exhibited severe hydropic degeneration and necrosis (Figure 5B, Table 2. In rats receiving erlotinib in combination with Liv-52, hydropic degeneration and necrosis were markedly attenuated and classified as mild (Figure 5C, Table 2). In the group applied with erlotinib and melatonin, hydropic degeneration and necrosis were observed at a moderate level of severity (Figure 5D, Table 2).

## 3. Discussion

In the current study, the effects of Liv-52 on erlotinib-related liver injury in rats were investigated using biochemical, histopathological, and immunofluorescence approaches and were evaluated in comparison with melatonin. Erlotinib is primarily metabolised by hepatic cytochrome enzymes, a process that has been suggested to render the liver particularly susceptible to drug-induced toxicity. Consistent with this notion, numerous case reports, clinical studies, and meta-analyses have documented erlotinib-associated hepatotoxicity [7,25,26,27]. However, preclinical studies addressing erlotinib-induced hepatotoxicity remain relatively limited in the literature [28]. In the present study, erlotinib administration resulted in a marked increase in MDA levels in rat liver tissues, accompanied by a reduction in antioxidant defense parameters, including tGSH, SOD, and CAT. In parallel, significant elevations in serum ALT, AST, and LDH activities were observed in the erlotinib group. An increase in apoptotic markers (PARP-1, AIF) has been observed with erlotinib use. Interestingly, erlotinib caused an increase in both inflammatory (COX-2) and anti-inflammatory (IL-10) parameters in liver tissues. Consistent with the biochemical findings, histopathological examination also corroborated the hepatotoxic effects induced by Erlotinib in liver tissues.

As is well established, one of the most significant indicators of oxidative stress in tissues is an increase in MDA levels. MDA is considered to be a final toxic product of lipid peroxidation (LPO) [29]. Following erlotinib administration, MDA levels in rat liver tissues showed a significant increase, according to the results of the current investigation. Consistent with these findings, previous experimental studies have shown that erlotinib induces systemic oxidative stress, leading to enhanced lipid peroxidation [30]. Similarly, a study investigating the hepatotoxic effects of gefitinib and afatinib reported increased MDA levels following EGFR-TKI administration [29]. In the present study, co-administration of Liv-52 with erlotinib significantly attenuated the erlotinib-related increase in hepatic MDA levels. Consistent with this finding, previous studies have also reported that Liv-52 protects cells against LPO [31,32]. In a study by Ciftel and colleagues, Liv-52 was shown to prevent pyrazinamide-induced increases in hepatic MDA production [20]. In the present study, Liv-52 suppressed the erlotinib-induced elevation in MDA levels more effectively than melatonin. Similarly, a previous study directly comparing Liv-52 and melatonin reported a greater reduction in MDA levels with Liv-52 treatment [20].

In the present study, hepatic tGSH, SOD, and CAT were also examined in erlotinib-treated animals, as oxidative stress is closely associated with decreased antioxidant capacity. When the ROS generated cannot be adequately scavenged by antioxidant systems, the balance shifts toward a pro-oxidant state, resulting in tissue injury [33,34]. In parallel with the increase in MDA levels, our results demonstrated a concomitant decrease in hepatic GSH, SOD, and CAT in the erlotinib group. GSH is one of the principal endogenous antioxidants involved in the maintenance of cellular redox balance and the neutralization of free radicals [8,33]. SOD and CAT are fundamental antioxidant enzymes that function sequentially and complementarily in the detoxification of ROS. During the reaction catalyzed by SOD, superoxide radicals are scavenged and converted into the less harmful hydrogen peroxide. This hydrogen peroxide is subsequently detoxified by CAT, thereby preventing its toxic accumulation [33]. The literature indicates that erlotinib is primarily metabolized in the liver via CYP3A4, and that reactive quinone–imine intermediates generated during this process may lead to GSH depletion [35]. Nevertheless, no preclinical studies evaluating the effects of erlotinib on antioxidant defense systems in liver tissues could be identified. In contrast, it has been reported that gefitinib, another EGFR tyrosine kinase inhibitor, reduces hepatic GSH levels and SOD activity. In the same investigation, CAT activity was slightly decreased in the gefitinib group relative to the control group, yet the change was not statistically meaningful [36]. In contrast to our findings, it has been reported that erlotinib prevents adenine-related increases in MDA levels and decreases in SOD and CAT activities in rat kidney tissues, suggesting a nephroprotective effect [37]. This apparent discrepancy may be attributable to the increased susceptibility of hepatic tissue to toxicity arising from reactive intermediate metabolites generated during erlotinib metabolism [25,35]. In the present study, both Liv-52 and melatonin significantly attenuated the erlotinib-related reductions in hepatic GSH levels and SOD and CAT activities. Notably, antioxidant defenses were more effectively preserved in the Liv-52-treated group than in the melatonin group. Numerous previous studies have demonstrated that the Liv-52 formulation prevents antioxidant depletion and significantly reduces oxidative damage [20,31]. A recent study also reported that Liv-52 suppressed hydroxychloroquine-induced antioxidant depletion in liver tissues [34]. Similarly, melatonin has been shown to limit oxidative damage by supporting endogenous antioxidant defense systems in various models of hepatotoxicity [38].

In the present study, serum ALT, AST, and LDH activities were also assessed. The literature reports that EGFR-TKI-induced damage is predominantly hepatocellular in nature and is accompanied by elevated transaminases [35]. Increased ALT and AST activities are widely accepted indicators of hepatocellular damage [39]. Although LDH is not a liver-specific biomarker, its activity may increase in the presence of hepatic injury [39]. The results of a meta-analysis conducted by Wu and colleagues showed that the increase in ALT and AST levels in patients using erlotinib was significant [27]. The present data showed that serum ALT, AST, and LDH activities were markedly elevated in the erlotinib group. In contrast, erlotinib-associated elevations in these enzymes were markedly attenuated in the groups receiving Liv-52 or melatonin. Notably, Liv-52 treatment resulted in a more pronounced improvement in serum enzyme activities compared with melatonin. Consistent with these findings, the hepatoprotective effects of Liv-52, including its ability to improve ALT and AST activities, have been documented in numerous clinical and preclinical studies [31]. Yildirim and colleagues showed that Liv-52 significantly inhibited doxorubicin-induced increases in ALT, AST, and LDH activities, as well as the associated tissue damage [40]. Similarly, exogenous melatonin treatment has been reported to attenuate hepatotoxicity by resulting in a milder elevation of hepatic biomarkers [41]. 

In this study, PARP-1 and AIF immunopositivity in liver tissues was evaluated using the immunofluorescence staining method. Previous studies have demonstrated that erlotinib induces PARP activation in hepatocytes and triggers apoptosis via the mitochondrial pathway [12]. PARP-1 is a member of the PARP enzyme family and plays a critical role in the DNA damage response. In cases of severe DNA damage, excessive activation of PARP-1 leads to disruption of the mitochondrial membrane potential, followed by the translocation of AIF from the mitochondria to the nucleus, resulting in DNA fragmentation, chromatin condensation, and ultimately cell death [42]. In this study, the absence of PARP-1 and AIF immunopositivity in healthy rats and their strong expression in the erlotinib-treated group suggest that erlotinib induces apoptosis signaling pathways [10]. However, in terms of PARP-1 and AIF, mild immunopositivity was detected in the Liv-52 group and moderate immunopositivity in the melatonin group. No information was found in the literature examining the effect of Liv-52 on PARP-1 activation. However, melatonin has previously been tested in acetaminophen-induced liver injury and has been reported to reduce hepatic nuclear AIF translocation [43].

In this study, to evaluate the effects of treatment protocols on the hepatic inflammatory response and the associated anti-inflammatory regulatory mechanisms, the immunopositivity of pro-inflammatory (COX-2) and anti-inflammatory (IL-10) markers was assessed in liver tissues using immunofluorescence staining [44,45]. Our findings revealed a significant increase in COX-2 immunopositivity in the liver tissues of the erlotinib group. COX-2 is an inducible enzyme that is upregulated in response to increased reactive oxygen species (ROS) production and inflammatory stimuli [44]. Although our literature survey did not identify direct evidence regarding the effect of erlotinib on COX-2, elevated COX-2 levels have been reported in various models of inflammatory liver injury, which is consistent with the marked COX-2 immunopositivity observed in the erlotinib group [46]. In contrast, Liv-52 administration significantly suppressed the erlotinib-induced increase in hepatic COX-2 immunopositivity. Although direct evidence regarding the effect of Liv-52 on COX-2 is limited, its hepatoprotective properties have been largely attributed to its anti-inflammatory and antioxidant activities [31]. Similarly, melatonin treatment markedly attenuated the increase in COX-2. Previous studies have demonstrated that melatonin exerts anti-inflammatory effects by inhibiting experimentally induced COX-2 in lung tissues [47]. In addition, IL-10 immunopositivity in liver tissues was evaluated in the present study. IL-10 is a potent anti-inflammatory cytokine that plays a pivotal role in immune regulation by suppressing macrophage activation and inhibiting the production of pro-inflammatory cytokines [45]. Interestingly, a significant increase in IL-10 immunopositivity was observed in the erlotinib group. In contrast to our findings, previous studies have reported that erlotinib reduces IL-10 expression in cancer cell lines [48], whereas a clinical study demonstrated that erlotinib treatment does not significantly alter circulating IL-10 levels [49]. Furthermore, in a doxorubicin-induced liver injury model, increased COX-2 immunopositivity was accompanied by decreased IL-10 levels [50]. However, in a recently conducted experimental study using an acute peritonitis model, IL-10 levels were also reported to increase in parallel with pro-inflammatory cytokines [45]. In the literature, increased IL-10 immunopositivity has been widely recognized as a compensatory feedback mechanism in response to tissue injury and inflammation [51]. Moreover, prostaglandin E2 derived from COX-2 has been shown to enhance IL-10 production, whereas inhibition of COX-2 results in decreased IL-10 levels [52]. Taken together, the observed increase in IL-10 immunopositivity in the present study is more likely to reflect a compensatory response aimed at limiting erlotinib-induced inflammatory damage rather than indicating a pro-inflammatory role. Liv-52 and melatonin-treated groups, the reduction in inflammatory markers was accompanied by a parallel decrease in IL-10 immunopositivity. This decline in IL-10 immunopositivity, together with the suppression of COX-2, may be interpreted as a consequence of reduced inflammatory burden, thereby eliminating the need for compensatory anti-inflammatory regulation.

The histopathological evaluation results in our study were consistent with the biochemical findings and double immunofluorescence staining data. Our results showed that erlotinib treatment induced severe hydropic degeneration and necrosis in liver tissues. In contrast, rats receiving Liv-52 in combination with erlotinib exhibited only mild tissue damage. Previous studies have similarly reported that Liv-52 significantly alleviates doxorubicin-induced liver injury [40]. In rats treated with melatonin, hydropic degeneration and necrosis persisted but were reduced to moderate levels. The literature also reports that exogenous melatonin treatment attenuates pathological changes in tissues in the context of hepatotoxicity [41].

The present study has several limitations that should be acknowledged. First, the evaluation of inflammatory and anti-inflammatory cytokines in liver tissue was not performed, which could have provided additional mechanistic insight into the hepatoprotective effects observed. Second, the combined effects of Liv-52, melatonin, and erlotinib were not investigated, representing an important area for future research. In addition, the assessment of oxidative stress was limited to a selected number of biomarkers, and molecular-level analyses such as gene or protein expression related to oxidative stress, inflammation, and apoptosis were not conducted.

## 4. Materials and Methods

### 4.1. Experimental Animals

The experimental animals consisted of 24 male Wistar albino rats (10–12 weeks of age; body weight 280–290 g). Animals were obtained from the Erzincan Binali Yildirim University Laboratory Animal Application and Research Center. At baseline, the rats were distributed randomly into four groups and kept under standard laboratory conditions for one week to allow adaptation (*n* = 6 per group). Each group was housed in a separate conventional cage (59 × 38 × 20 cm; floor area: 2240 cm^2^). All rats were kept in a controlled environment, featuring a 12 h light/dark cycle, a consistent temperature of 22 ± 2 °C, and relative humidity levels of 40–70%. During both the acclimatization period and the experimental procedures, the rats had ad libitum access to tap water and standard laboratory chow (Bayramoğlu Anonim Şirketi, Erzurum, Turkey). All procedures were designed to minimize animal suffering and to use the smallest number of animals necessary to obtain scientifically reliable results. This study was carried out in compliance with the European Directive 2010/63/EU on the protection of animals used for scientific purposes (Approval ID: 2016-24-199), and the study design and reporting adhered to the ARRIVE (Animal Research: Reporting of In Vivo Experiments) guidelines [53].

### 4.2. Chemical Substances

Erlotinib (Tarceva^®^, 100 mg tablet) was sourced from Roche (Istanbul, Turkey), Liv-52 (Liv.52^®^ tablet) was purchased from Himalaya Wellness Company (Bengaluru, India), Melatonin (Melatonina LEK-AM^®^, 3 mg tablet) was supplied by Lek-AM (Zakroczym, Warsaw, Poland), and thiopental sodium (Pental Sodium^®^, 0.5 g powder for injection) was supplied by Menarini (Istanbul, Turkey).

### 4.3. Experimental Design and Randomization

To adhere to the 4R (Reduction, Refinement, Replacement, and Responsibility) principles and obtain reproducible, scientifically sound results, the smallest necessary number of animals was selected [54]. Exclusion criteria were predefined as pre-experimental or during/post-experimental. Prior to the study, animals were evaluated for atypical posture, decreased activity levels, or the presence of injuries. During/post-experimental criteria encompassed unexpected death, complications associated with anesthesia or drug administration, procedural errors, any deviations from the protocol or incomplete dosing of study agents, body weight loss >15–20%, systemic illness or severe distress, and compromised tissue integrity affecting biochemical or histopathological analyses. No rats fulfilled the exclusion conditions; all were included in the final analyses. To minimize bias, each animal and cage was assigned a unique identification code maintained throughout the study. All evaluations were performed by researchers blinded to group distribution.

### 4.4. Animal Groups

The rats were randomly assigned into four groups (*n* = 6 per group): Healthy group (HG), erlotinib group (ERG), Liv-52 + erlotinib group (LEG), and melatonin + erlotinib group (MEG).

### 4.5. Experimental Procedure

**Figure 6:**
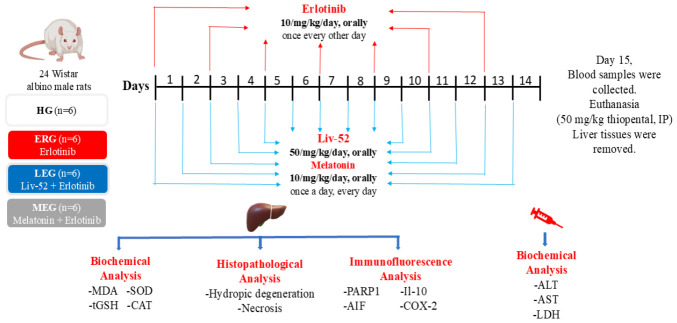
Experimental design scheme. MDA: malondialdehyde; tGSH: total glutathione; SOD: superoxide dismutase; CAT: catalase; ALT: alanine aminotransferase; AST: aspartate aminotransferase; LDH: lactate dehydrogenase; PARP-1: poly(ADP-ribose) polymerase 1; AIF: apoptosis-inducing factor; IL-10: interleukin 10; COX-2: cyclooxygenase 2; IP: intraperitoneally; HG: healthy group; ERG: erlotinib group; LEG: Liv-52 + erlodipine group; MEG: melatonin + erlotinib group.

As outlined in Figure 6, Liv-52 (50 mg/kg) [40] was applied orally by gastric gavage to the LEG group, while melatonin (10 mg/kg) [20] was given to the MEG group using the same route. Concurrently, an equivalent volume of pure water was applied orally to the HG and ERG groups. One hour post-administration of Liv-52, melatonin, or plain water, erlotinib (10 mg/kg) was orally delivered to rats in the ERG, LEG, and MEG groups [8]. Throughout this period, the HG group was orally provided with an equivalent volume of pure water. The experimental protocol was conducted for 14 days, during which erlotinib was given every other day, whereas Liv-52 and melatonin were given once daily. On day 15, blood samples were collected from the tail veins of the rats for the determination of serum alanine aminotransferase (ALT), aspartate aminotransferase (AST), and lactate dehydrogenase (LDH) activities. Subsequently, euthanasia was performed using intraperitoneal thiopental sodium (50 mg/kg), and liver tissues were excised. In the excised liver tissues, MDA, tGSH, SOD, and CAT were evaluated. Moreover, histopathological and immunofluorescent examinations were performed on liver tissues by a pathologist who was unaware of the trial groups.

### 4.6. Biochemical Analyses

#### 4.6.1. Liver Tissue MDA, tGSH, SOD, and CAT Analyses

Liver tissue samples (0.2 g) were accurately weighed and rinsed with physiological saline. Tissue specimens were rapidly cryopreserved in liquid nitrogen and homogenized without delay. The resulting homogenates were transferred into a 50 mM buffer solution (pH 7.2) and subsequently centrifuged (3000 rpm, 20 min, 4 °C). The clarified fraction was collected and retained at −80 °C until further biochemical analyses.

MDA (Cat. No. E0156Ra), GSH (Cat. No. E1101Ra), and SOD (Cat. No. E0168Ra) levels in liver tissue were measured using rat-specific enzyme-linked immunosorbent assay (ELISA) kits obtained from Assay BT LAB (Hangzhou, China). CAT levels were quantified using a rat ELISA kit purchased from Rel Assay Diagnostics (Mega Tıp, Gaziantep, Turkey). All biochemical analyses were performed in accordance with the manufacturers’ instructions.

#### 4.6.2. Measurement of Serum ALT, AST, and LDH Activities

Whole blood samples were obtained using anticoagulant-free tubes and incubated at room temperature until coagulation was complete. The specimens were then centrifuged (3000 rpm, 10 min), after which the serum portion was carefully recovered and maintained at −80 °C before biochemical evaluation.

Serum levels of ALT, AST, and LDH were analyzed using an automated AU5800 chemistry analyzer (Beckman Coulter, Brea, CA, USA), applying kinetic UV methodologies consistent with the standards set by the International Federation of Clinical Chemistry and Laboratory Medicine. The assays rely on monitoring the rate of nikotinamid adenin dinükleotid oxidation at 340 nm in coupled enzymatic reactions, and the decrease in absorbance was directly proportional to the enzyme activities.

### 4.7. Double Immunofluorescence Method

Paraffin-embedded liver tissue sections (5 μm) mounted on poly-L-lysine-coated slides were deparaffinized in xylene, rehydrated through a graded alcohol series, and washed with phosphate-buffered saline (PBS). Antigen retrieval was performed using citrate buffer (pH 7.4) at 800 W for two cycles of 5 min. Sections were then washed twice with PBS for 10 min each. The sections were incubated in PBS/gelatin/Triton X-100 (0.25%) solution for 10 min. Blocking was then performed with 5% BSA for 1 h. Then, the sections were incubated overnight at 4 °C with the following primary antibody pairs at a dilution of 1:200: monoclonal PARP-1 (Santa Cruz Biotechnology, Cat. No. sc-74470, Lot: G0325) with polyclonal AIF (Bioss [Beijing, China], Cat. No. bs-0037R, Lot: AD091507), and monoclonal IL-10 (Santa Cruz Biotechnology [Dallas, TX, USA], Cat. No. sc-8438, Lot: K1613) with polyclonal COX-2 (BT-Lab [Hangzhou, China], Cat. No. BT-AP08027, Lot: MFG20241022). After washing again with PBS, the sections were incubated for 45 min with a secondary antibody cocktail prepared in 1% BSA, containing goat anti-mouse FITC (Jackson ImmunoResearch [West Grove, PA, USA], Cat. No. 115-095-003) and anti-rabbit Alexa Fluor 595 (Cell Signaling Technology [Danvers, MA, USA], Cat. No. 8889S), mixed in equal amounts and used at a dilution of 1:100. The sections were subsequently washed first with PBS and then with 10 mM CuSO_4_/50 mM NH_4_Cl solution for 10 min. Following washing with distilled water, DAPI (4′,6-diamidino-2-phenylindole) was applied to the sections, which were then examined using a fluorescence microscope system (Zeiss Axiolab 5, Jena, Germany) equipped with an Axiocam 305 color camera and Colibri 3 light source. During evaluation, AIF and COX-2 immunopositivity were visualized in red, whereas PARP-1 and IL-10 immunopositivity were visualized in green and assessed using Zen Blue 3.1 software as absent (0), mild (1), moderate (2), severe (3), or very severe (4). Given the potential limitations of automated analysis due to autofluorescence, all evaluations were performed independently by two experienced pathologists in a blinded manner, and discrepancies were resolved by consensus.

### 4.8. Histopathological Evaluation

For histological examination, liver tissues were fixed in 10% neutral buffered formalin. The fixed samples were washed in flowing tap water for 24 h, dehydrated using increasing concentrations of ethanol, rendered transparent in xylene, and embedded in paraffin wax. Sections measuring 5 μm were prepared from the paraffin blocks and stained with hematoxylin–eosin. Histopathological evaluation was carried out on a total of 24 rats, with six animals in each experimental group. For each rat, one central and five peripheral fields were systematically selected from serial tissue sections for microscopic assessment. Microscopic evaluation was performed in a blinded manner by a pathologist using an Olympus BX51 light microscope, and representative fields were documented using an Olympus DP71 digital camera (Tokyo, Japan). Liver sections were examined for hydropic degeneration and necrosis. The severity of histopathological damage was semi-quantitatively scored on a scale from 0 to 3, where 0 indicates no damage, 1 mild, 2 moderate, and 3 severe [55].

### 4.9. Statistical Analyses

All data analyses were carried out with IBM SPSS Statistics (v22.0; Armonk, NY, USA). Graphical illustrations were produced with GraphPad Prism software (v. 9.0.0; San Diego, CA, USA). Biochemical parameters are expressed as mean ± SD. The Shapiro–Wilk test was performed to examine the distribution of the data for normality. Based on these findings, one-way ANOVA was used to compare groups. The assumption of variance homogeneity was tested using Levene’s test. Since homogeneity of variances was confirmed, pairwise group comparisons were performed using Tukey’s Honestly Significant Difference test. Non-parametric semi-quantitative histopathological and immunofluorescence data were analyzed with the Mann–Whitney U test and showed as median (25th–75th percentile). A threshold of *p* < 0.05 was accepted as statistically significant.

## 5. Conclusions

In the present study, the hepatoprotective effects of Liv-52 and melatonin were comparatively evaluated in erlotinib-induced liver injury in rats. Erlotinib administration increased MDA levels and decreased tGSH levels, as well as SOD and CAT enzyme activities, in liver tissue. Serum ALT, AST, and LDH activities were also elevated. Moreover, erlotinib induced significant histopathological damage in the liver. In addition, marked immunopositivity was observed for apoptotic markers (PARP-1, AIF) and the inflammatory marker (COX-2), as well as the anti-inflammatory marker (IL-10). Although both Liv-52 and melatonin exerted mitigating effects against erlotinib-induced hepatotoxicity, the protective response appeared to be more pronounced in the Liv-52-treated group. These findings suggest that Liv-52 may represent a potential candidate for preserving liver function and tissue integrity during erlotinib therapy.

## Figures and Tables

**Table 1:** Immunofluorescence-detected PARP-1, AIF, IL-1, and COX-2 immunopositivity in liver tissues.

Parameters	Experimental Groups (*n* = 6/Each Group)			
	HG	ERG	LEG	MEG
	Median (25th–75th Percentile)			
PARP-1	0 (0–0)	3 (2.75–3.25) *	1 (0.75–1.25) *^,#^	2 (1.75–2.25) *^,#^
AIF	0 (0–0)	3 (2.75–4) *	1 (0.75–1.25) *^,#^	1.5 (1–3) *^,#^
IL-10	0 (0–0)	4 (3.75–4) *	1 (1–1.25) *^,#^	3 (2–3.25) *^,#^
COX-2	0 (0–0)	4 (3–4) *	2 (1–2) *^,#^	2.5 (2–3.25) *^,#^

Immunopositivity: 0; absent, 1; mild, 2; moderate; 3; severe, 4; extremely severe. *; *p* < 0.05 vs. HG, ^#^; *p* < 0.05 vs. ERG. PARP-1: poly (ADP-ribose) polymerase 1; AIF: apoptosis-inducing factor; IL-10: interleukin 10; COX-2: cyclooxygenase 2; HG: healthy group; ERG: erlotinib group; LEG: Liv-52 + erlotinib group; MEG: melatonin + erlotinib group. Statistical analysis was performed by Mann–Whitney U test. *p* < 0.05 was considered significant.

**Table 2:** Histopathological damage scores in liver tissues.

Parameters	Experimental Groups (*n* = 6/Each Group)			
	HG	ERG	LEG	MEG
	Median (25th–75th Percentile)			
Hydropic degeneration	0 (0–0)	3 (2–3) *	1 (0–1) *^,#^	1.5 (1–2.25) *^,#^
Necrosis	0 (0–0)	3 (2.75–3) *	1 (0.75–1.25) *^,#^	2 (1.75–2.25) *^,#^

Histopathological damage scoring: 0; absent, 1; mild, 2; moderate; 3; severe. *; *p* < 0.05 vs. HG, ^#^; *p* < 0.05 vs. ERG. HG: healthy group; ERG: erlotinib group; LEG: Liv-52 + erlodipine group; MEG: melatonin + erlotinib group. Statistical analysis was performed by Mann–Whitney U test. *p* < 0.05 was considered significant.

## Data Availability

The original contributions presented in this study are included in the article. Further inquiries can be directed to the corresponding author.
