# Correction: Bulut et al. Liv-52 Attenuates Erlotinib-Induced Liver Injury via Modulation of Oxidative Stress, Inflammation, and Apoptosis in Rats. *Int. J. Mol. Sci.* 2026, *27*, 3817

**DOI:** 10.3390/ijms27219554

**Published:** 2026-10-26

**Authors:** Seval Bulut, Durdu Altuner, Bahadir Suleyman, Renad Mammadov, Mustafa Ozkaraca, Ali Gungor, Mehmet Kuzucu, Engin Hendem, Halis Suleyman

**Affiliations:** 1 Department of Pharmacology, Faculty of Medicine, Erzincan Binali Yıldırım University, Erzincan 24100, Turkey; daltuner@erzincan.edu.tr (D.A.); bsuleyman@erzincan.edu.tr (B.S.); rmammadov@erzincan.edu.tr (R.M.); hsuleyman@erzincan.edu.tr (H.S.); 2 Department of Pathology, Faculty of Veterinary Medicine, Sivas Cumhuriyet University, Sivas 58070, Turkey; mustafaozkaraca@cumhuriyet.edu.tr; 3 Laboratory Technician and Veterinary Health Program, Vocational School of Health Services, Osmaniye Korkut Ata University, Osmaniye 80000, Turkey; aligungor@osmaniye.edu.tr; 4 Department of Molecular Biology, Faculty of Arts and Sciences, Erzincan Binali Yıldırım University, Erzincan 24100, Turkey; mkuzucu@erzincan.edu.tr; 5 Department of Medical Oncology, Mengucek Gazi Education and Research Hospital, Erzincan Binali Yıldırım University, Erzincan 24100, Turkey; engin.hendem@saglik.gov.tr

The following reference [47] has been retracted and should be removed from the original publication [1]:

[47]. Cuzzocrea, S.; Costantino, G.; Mazzon, E.; Caputi, A.P. Regulation of prostaglandin production in carrageenan-induced pleurisy by melatonin. *J. Pineal Res.* **1999**, *27*, 9–14.

The scientific content of [47] can be replaced with the following reference:

Atasever, A.; Tekin, S.; Bolat, İ.; Bolat, M.; Dağ, Y.; Çınar, B.; Şengül, E.; Yıldırım, S.; Warda, M.; Çelebi, F. The effects of melatonin on oxidative stress, inflammation, apoptosis and Nrf2/HO-1 in acrylamide-induced lung injury in rats. *Naunyn. Schmiedebergs Arch. Pharmacol.* **2025**, *398*, 15905–15922.

The authors state that the scientific conclusions are unaffected. The corrections were approved by the Academic Editor. The original publication has also been updated.
